# The Complexity of Standing Postural Sway Associates with Future Falls in Community-Dwelling Older Adults: The MOBILIZE Boston Study

**DOI:** 10.1038/s41598-017-03422-4

**Published:** 2017-06-07

**Authors:** Junhong Zhou, Daniel Habtemariam, Ikechukwu Iloputaife, Lewis A. Lipsitz, Brad Manor

**Affiliations:** 1 Hebrew SeniorLife Institute for Aging Research, Roslindale, MA USA; 2 Beth Israel Deaconess Medical Center, Boston, MA USA; 3 Harvard Medical School, Boston, MA USA

## Abstract

Standing postural control is complex, meaning that it is dependent upon numerous inputs interacting across multiple temporal-spatial scales. Diminished physiologic complexity of postural sway has been linked to reduced ability to adapt to stressors. We hypothesized that older adults with lower postural sway complexity would experience more falls in the future. 738 adults aged ≥70 years completed the Short Physical Performance Battery test (SPPB) test and assessments of single and dual-task standing postural control. Postural sway complexity was quantified using multiscale entropy. Falls were subsequently tracked for 48 months. Negative binomial regression demonstrated that older adults with lower postural sway complexity in both single and dual-task conditions had higher future fall rate (incident rate ratio (IRR) = 0.98, p = 0.02, 95% Confidence Limits (CL) = 0.96–0.99). Notably, participants in the lowest quintile of complexity during dual-task standing suffered 48% more falls during the four-year follow-up as compared to those in the highest quintile (IRR = 1.48, p = 0.01, 95% CL = 1.09–1.99). Conversely, traditional postural sway metrics or SPPB performance did not associate with future falls. As compared to traditional metrics, the degree of multi-scale complexity contained within standing postural sway-particularly during dual task conditions- appears to be a better predictor of future falls in older adults.

## Introduction

Older adults commonly fall due to loss of balance when standing^1^. The task of standing is most often completed as part of a “dual task;” that is, standing while simultaneously performing additional cognitive tasks such as talking, reading or making decisions in daily life^1^. Such dual tasking often interferes with performance in one or both tasks, especially in older adults^2^. Numerous attempts have thus been made to identify fall risk by measuring one’s ability to regulate the movement of their body’s center of mass (i.e., postural sway) with respect to its base of support, under both normal and dual task conditions^3^. Older adults with a history of falls tend to have larger and faster postural sway when standing in either of these conditions, as compared to those who have not fallen in the past^4^. However, such traditional characterizations of postural sway, which tend to characterize sway motion or structure at a single temporal or spatial scale (e.g., average sway speed or area) do not sensitively predict those older adults who are more likely to fall in the future^5–7^. For example, in a study of 100 older adults reported by Maki *et al*.^7^, baseline spatial characteristics of postural sway, including average sway speed, did not correlate with prospective fall rates in the ensuing twelve-month follow-up period. We contend that the inability of traditional postural sway metrics to predict future falls may be due to the insensitivity of these metrics to the complex nature of the postural control system.

The regulation of one’s standing postural sway requires the integration of numerous sensory inputs, spinal and supraspinal circuits, a host of cognitive functions and the peripheral neuromuscular system, all operating over different time scales^8^. Consequently, this control system is inherently non-linear. Characteristic of a non-linear system, the relationship between these inputs and related muscular outputs is continuously modulated over time; the system dynamically “re-weights” the relative influence of each type of feedback on postural muscle activation in order to optimize performance in a given situation^9, 10^. Similarly, the amount of joint torque created by a given muscular contraction is dependent upon joint stiffness, which is also continuously modulated via changes in muscular tone^11^. As a result of these and other inter-related and ever-changing control strategies, the seemingly spontaneous fluctuations of postural sway are actually “complex,” meaning that they contain a degree of correlated, fractal-like patterns that exhibit self-similar structures across multiple scales of time and/or space^12–15^. A previous study has demonstrated that the degree of postural sway complexity is largely independent of traditional sway metrics such as average sway speed or area over time^16^. We therefore hypothesized that as opposed to traditional metrics, those metrics aimed at capturing the physiologic “complexity” of postural sway will more accurately reflect the integrity of the postural control system and thus, better identify those at risk of suffering falls in the future.

In this study, we conducted a secondary analysis of longitudinal data from the population-based MOBILIZE Boston Study^17^ to determine the relationship between postural sway and future falls in community-dwelling older adults. Here, we chose to quantify the degree of postural sway complexity during single- and dual-task standing using a technique called multiscale entropy (MSE)^18^. MSE is one of numerous non-linear time-series analytical techniques that have been used to estimate postural sway complexity^19^. This approach quantifies the degree of re-occurrence of repetitive patterns within sway fluctuations. However, as opposed to traditional entropy analyses that are limited to one single time scale (e.g., approximate entropy or sample entropy), MSE utilizes a “coarse-graining” technique to estimate the degree of entropy contained within the time series across multiple scales of time. We specifically hypothesized that that those with lower MSE-derived complexity of postural sway during single or dual task standing at baseline would suffer more falls in the future.

## Results

765 participants completed baseline tests of postural sway and the Short Physical Performance Battery test (SPPB). 738 of these individuals completed 48 consecutive months of falls tracking. Analyses were limited to these 738 participants. Baseline clinical and functional characteristics of fallers (i.e., those who fell at least once in 48 months) and non-fallers in this cohort are listed in Table 1. The self-reported historical falls rate was higher in fallers than non-fallers (p < 0.001), while all other health characteristics were similar between those who did and did not fall during the study period.

**Table 1:** Demographics of fallers and non-fallers.

	Total participants	Fallers (n = 460)	Non-fallers (n = 278)	p-value*
Age (years), Mean ± SD	78.1 ± 5.4	78.2 ± 5.5	77.9 ± 5.3	0.43
Female, n (%)	470 (64)	292 (63)	178 (64)	0.79
BMI, Mean ± SD	27.3 ± 5.1	27.3 ± 4.9	27.4 ± 5.4	0.64
Education (years), Mean ± SD	14.2 ± 3.1	15.0 ± 4.8	14.6 ± 7.7	0.11
Comorbidity, Mean ± SD	3.0 ± 1.6	3.1 ± 1.6	2.9 ± 1.6	0.32
SPPB, Mean ± SD	9.3 ± 2.5	9.3 ± 2.6	9.4 ± 2.4	0.48
Historical falls rate	0.7 ± 1.3	0.9 ± 1.5	0.3 ± 0.7	<0.001
Sway speed (mm/s), Mean ± SD				
ST	19.1 ± 4.9	19.3 ± 4.9	18.9 ± 4.8	0.39
DT	21.5 ± 6.9	21.7 ± 7.4	21.3 ± 6.1	0.43
Sway area (mm^2^/s), Mean ± SD				
ST	183.3 ± 140.7	191.1 ± 156.3	170.5 ± 109.2	0.24
DT	236.4 ± 217.6	248.0 ± 240.3	217.1 ± 172.2	0.29
AP path length (mm), Mean ± SD				
ST	447.1 ± 124.6	448.9 ± 123.0	443.9 ± 127.4	0.59
DT	506.8 ± 179.1	509.7 ± 191.0	502.1 ± 157.5	0.58
Complexity, Mean ± SD				
ST	31.2 ± 6.3	30.7 ± 6.4	31.9 ± 6.2	0.007
DT	31.0 ± 7.5	30.4 ± 7.6	32.1 ± 7.3	0.002

*ANOVAs and Chi-Square test (for sex) were used to determine the differences in these characteristics between fallers and non-fallers.

Note: BMI = body mass index; ST = single task standing; DT = dual task standing; AP = anterioposterior.

When comparing the postural sway metrics, fallers exhibited lower postural sway complexity in both ST and DT conditions (p < 0.007) at baseline than non-fallers, while the traditional parameters (i.e., sway speed, sway area and anterioposterior (AP) path length) or SPPB score did not differ between fallers and non-fallers.

### Relationship between baseline standing postural sway and future falls

**Figure 1:**
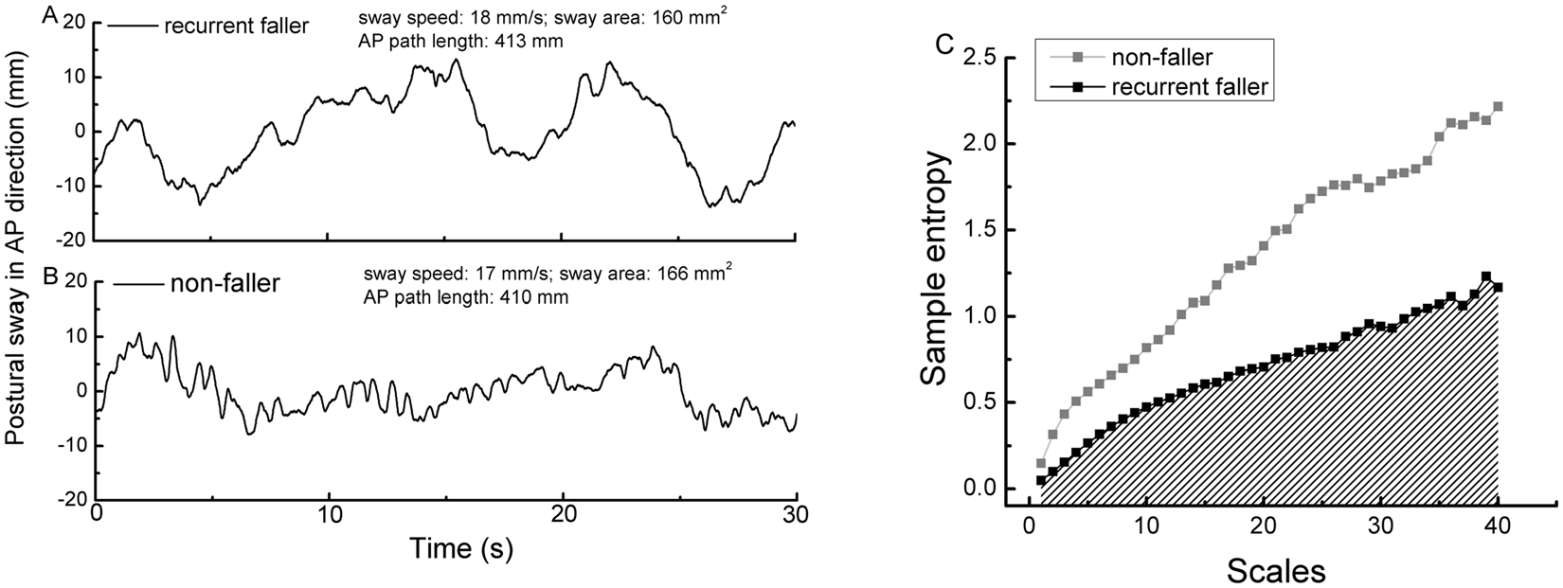
Illustrative anterioposterior (AP) postural sway time-series of a recurrent faller (**A**) and a non-faller (**B**) during single task quiet standing along with multiscale entropy (MSE) curves generated from each time-series (**C**). To quantify the different postural sway dynamics of the time series in A and B, sample entropy was calculated and plotted as a function of time scales (ranging from scale 1 to 40) for each time-series. Postural sway complexity was defined as the area under the multiscale entropy curve, as illustrated by gray shading under the curve of the recurrent faller. When compared to the non-faller, sample entropy of this recurrent faller was lower across multiple time scales. Postural sway complexity (i.e., area under the multiscale entropy curve) of this recurrent faller (complexity = 27.5 units) was nearly half that of the non-faller (complexity = 52.8 units) while other postural sway metrics (i.e., sway speed, area and AP path length) of the two participants were similar (listed on the top of A and B).

Figure 1 shows the AP COP time-series during ST from a recurrent faller (A) and a non-faller (B), along with the MSE curves generated from each time-series (C). As compared to the non-faller, the sample entropy of the current faller was lower across multiple scales of time. As such, postural sway complexity (i.e., the area under the MSE curve) of the participant who experienced falls was considerably less than the participant who did not. In contrast, other metrics of postural sway, including sway speed, sway area and AP path length, were quite similar between these two study participants.

The associations between baseline postural sway metrics (i.e., complexity, sway speed, sway area, and AP path length) and the rate of future falls were analyzed using negative binomial regression analyses. Results indicated that the complexity of postural sway during ST (incident rate ratio (IRR) = 0.98, p = 0.02, 5% Confidence Limits (CL): 0.96–0.99) and DT (IRR = 0.98, p = 0.02, 95% CL: 0.97–0.99) was negatively associated with future falls rate (Table 2); one unit difference of either single or dual task postural sway complexity associated with 2% difference in future falls rate. In other words, participants with lower baseline postural sway complexity had a higher rate of future falls. These relationships were independent of age, sex, BMI and historical falls. Conversely, SPPB score or traditional postural sway measures (i.e., sway speed, area, and AP path length) did not significantly predicted the incidence of future falls (IRRs = 1~1.01, p > 0.49).

**Table 2:** Relationship between baseline metrics and the rate of future falls^#^.

		Total falls rate			
		IRR	p-value	95% CL	
Sway speed	ST	1.01	0.49	0.99	1.03
	DT	1	0.87	0.99	1.02
Sway area	ST	1	0.92	1	1.001
	DT	1	0.82	1	1.001
AP path length	ST	1	0.94	1	1.001
	DT	1	0.89	1	1.002
SPPB		1.01	0.76	0.96	1.05
Postural sway complexity	ST	0.98	0.02	0.96	0.99
	DT	0.98	0.02	0.97	0.99

^#^The negative binomial regression analyses were adjusted for age, sex, BMI and historical falls rate.

Note: ST = single task standing; DT = dual task standing; AP = anterioposterior; IRR = incident rate ratio; 95% CL: 95% Confidence Limits.

### Incident rate ratio of future falls in postural sway complexity quintiles

We also divided the cohort into quintiles of postural sway complexity, separately for both ST and DT conditions (Table 3). Participants in the “low-complexity” quintiles were younger those in than “high-complexity” quintiles (p < 0.001) (Table 4). Other health characteristics were similar between quintiles. The lower quintiles suffered higher rates of falls (p < 0.03). Post-hoc analyses revealed that: 1) older adults in Quintile 1 of complexity during ST had a significantly higher falls rate than those in Quintiles 4 and 5 (p < 0.01); and 2) those in Quintiles 1, 2 and 3 of complexity during DT had higher falls rate than those in Quintiles 4 and 5 (p < 0.04).

**Table 3:** Postural sway complexity quintiles.

	ST postural sway complexity			DT postural sway complexity		
	Mean ± SD	Range		Mean ± SD	Range	
Quintile 1	23.1 ± 2.5	14.8	26.2	21.3 ± 2.6	12.6	24.5
Quintile 2	27.6 ± 0.8	26.2	28.9	26.7 ± 1.2	24.5	28.6
Quintile 3	30.6 ± 1.0	28.9	32.2	30.5 ± 1.1	28.6	32.3
Quintile 4	33.9 ± 1.0	32.2	35.8	34.3 ± 1.3	32.3	37.1
Quintile 5	40.7 ± 3.9	35.7	53.2	42.3 ± 4.5	37.1	59.1

*Note*: ST = single task standing; DT = dual task standing.

**Table 4:** Descriptive characteristics of participants in quintiles of postural sway complexity.

		Age (years), Mean ± SD	BMI, Mean ± SD	Female, n (%)	Education (years), Mean ± SD	Comorbidity, Mean ± SD	Falls rate^#^, Mean ± SD
Single task standing	Quintile 1	77.8 ± 5.6^A^	27.6 ± 5.0	97 (66)	14.1 ± 3.5	3.0 ± 1.7	2.6 ± 3.6^A^
	Quintile 2	77.0 ± 4.9^A^	27.4 ± 4.8	99 (67)	14.6 ± 2.9	3.2 ± 1.6	2.1 ± 3.0^AB^
	Quintile 3	77.9 ± 5.7^AB^	26.8 ± 5.2	93 (63)	14.3 ± 2.9	3.0 ± 1.6	2.2 ± 2.8^AB^
	Quintile 4	78.3 ± 5.3^AB^	27.4 ± 5.1	97 (66)	14.3 ± 3.2	2.9 ± 1.5	1.8 ± 2.5^B^
	Quintile 5	79.6 ± 5.2^C^	27.4 ± 5.5	84 (57)	14.1 ± 2.9	3.1 ± 1.6	1.7 ± 2.4^B^
	p-value*	0.001**	0.69	0.39	0.79	0.65	0.03**
Dual task standing	Quintile 1	77.6 ± 5.7^A^	27.4 ± 4.6	98 (67)	14.1 ± 3.3	2.8 ± 1.7	2.4 ± 2.9^A^
	Quintile 2	76.9 ± 5.2^AB^	27.2 ± 5.1	103 (69)	14.2 ± 3.1	2.9 ± 1.4	2.5 ± 3.7^AB^
	Quintile 3	77.4 ± 5.1^A^	27.5 ± 4.9	90 (62)	14.6 ± 3.1	3.2 ± 1.7	2.1 ± 2.9^AB^
	Quintile 4	78.7 ± 5.4^AC^	27.5 ± 5.9	92 (62)	14.3 ± 2.9	3.3 ± 1.6	1.8 ± 2.5^BC^
	Quintile 5	80.2 ± 5.1^C^	27.0 ± 5.0	87 (59)	14.2 ± 3.0	2.9 ± 1.5	1.4 ± 2.0^C^
	p-value*	<0.0001**	0.94	0.37	0.65	0.07	0.009**

^#^Falls were tracked for 48 months by using self-reported calendar.

^*^ANOVAs and Student’s t post-hoc tests were used to determine the differences in these characteristics between quintiles. Within each column in ST and DT conditions, mean values with different superscript letters (A, B, or C) are significantly different from one another as determined by Student’s t post-hoc tests, P < 0.05.*Note*: BMI = body mass index.

Negative binomial regression analyses further indicated that as compared to the highest quintile of complexity (Quintile 5) derived from the ST condition, older adults in the lowest quintile of complexity (Quintile 1) exhibited a (non-significant) trend towards a higher rate of future falls (Table 5, IRR = 1.30, p = 0.07, 95% CL: 0.97–1.75). Notably, older adults in the lower quintiles of complexity (Quintile 1, 2 and 3) derived from the DT condition, however, experienced 42–48% more falls during the follow-up (Table 5, IRRs = 1.42~1.48, p < 0.03, 95% CLs: 1.04–1.99).

**Table 5:** Relationship between postural sway complexity quintiles and the rate of future falls^#^.

	Complexity in ST				Complexity in DT			
	IRR	p-value	95% CL		IRR	p-value	95% CL	
Quintile 1	1.30	0.07	0.97	1.75	1.48	0.01	1.09	1.99
Quintile 2	1.14	0.41	0.84	1.54	1.42	0.03	1.04	1.93
Quintile 3	1.13	0.43	0.83	1.53	1.44	0.02	1.06	1.97
Quintile 4	0.92	0.57	0.67	1.25	1.34	0.06	0.98	1.83
Quintile 5	Reference							

^#^The negative binomial regression analyses were adjusted for age, sex, BMI and historical falls rate. Quintile 5 was set as the reference quintile.

Note: ST = single task standing; DT = dual task standing; IRR = incident rate ratio; 95% CL: 95% Confidence Limits.

## Discussion

Falls are a major health concern for older adults because they often result in fractures, hospitalization, diminished mobility, and even death^20, 21^. Our results from a large cohort of community-dwelling older adults indicate that the multi-scale physiologic complexity of standing postural sway predicts falls in community-dwelling older adults, even after adjusting for age, BMI, sex, and the historical self-reported falls rate. In general, we observed that older adults with lower baseline complexity of AP postural sway when standing quietly or while performing a cognitive task, demonstrated higher fall rates over the ensuing 48 months. In contrast, commonly-used traditional metrics of postural sway, including sway speed, area and path length, or the physical function test (i.e., SPPB) were not predictive of future falls. Taken together, these results suggest that the MSE-derived complexity of standing postural sway provides unique insight into the complex nature of the postural control system that may be utilized to help identify those older adults who are more likely to fall in the future.

The behavior of a given physiological system is controlled by numerous inputs and regulatory elements that interact with one another on multiple scales of time and space^22^. The “complexity theory of aging” states that age-related alterations in the quantity and/or quality of these components, as well as to their structural and functional connectivity, reduce system functionality and impair an organism’s ability to adapt to stress. Importantly, mounting evidence across numerous physiological systems suggests that these changes also manifest in a reduction of the complexity contained within the dynamics of the system’s behavior or output under basal or “free-running” conditions^23–26^.

In line with this theory, lower complexity of standing postural sway has been linked to diminished quantity and/or quality of sensory “input” to the postural control system^16^. Manor *et al*.^16^ demonstrated that older adults with reduced ability to detect light pressures applied to the foot soles, or those with reduced visual acuity, had lower standing postural sway complexity as compared to their age-matched counterparts with intact sensory function. Moreover, those older adults with deficits in both sensory systems exhibited even lower sway complexity. Separate studies have also linked lower postural sway complexity to diminished capacity to adapt to stressors, as indicated by frailty^27^ and falls history^28^. Here, we have provided first-of-its-kind, prospective evidence that lower postural sway complexity at baseline is also independently associated with higher rates of future falls over a four-year follow up period.

In contrast to postural sway complexity, traditional metrics related to the average speed or magnitude of sway fluctuations did not predict future falls. The majority of balance-related falls stem from complex, multi-scale interactions between the individual, the environment, and the tasks being completed^29^. As such, traditional postural sway metrics, which reflect the dynamics or behavior of standing postural control on only a single scale of time, may not fully capture a person’s ability to adapt to the stressors of daily life and ultimately, avoid falls. This notion is supported by Fernie *et al*.^5^, who similarly reported that average standing postural sway speed did not correlate with the frequency of future falls in 205 older adults aged 80 years and above. This inability of traditional sway metrics to predict falls may stem from the idea that the dynamic characteristics of a healthy postural control states are not simply reflected as less or reduced sway variability^30^. To this end, Tai Chi training, for example, has been shown to improve mobility in multiple populations, and at the same time, increase the speed of postural sway fluctuations during standing^31–33^.

In the current study, those who fell during the follow-up period had lower baseline postural sway complexity, within both ST and DT conditions, as compared to those who did not suffer a fall. Interestingly, within the subset of the cohort that fell at least once, we observed that 1) average sway complexity was similar across ST and DT conditions, yet 2) the degree of postural sway complexity specifically derived from DT trials appeared to be a better predictor of the incidence of future falls. Negative binomial regression models demonstrated that participants within the lowest quintile of DT postural sway complexity, as compared to the highest quintile, suffered 48% more falls during the follow-up period. In contrast, there was only a marginal increase in future falls rate in the lowest quintile of ST postural sway complexity as compared to the highest quintile. The particular sensitivity of DT postural sway complexity to future falls may stem from the notion that the DT test condition more closely mimics typical situations in which falls occur; namely, when older adults are standing or walking and attempt to execute concurrent tasks such as speaking to others, reading or problem-solving^34–36^.

The current observation that those with higher postural sway complexity at baseline have lower incidence of future falls suggests that fall prevention strategies may be optimized by targeting the complex dynamics of postural control. Several studies suggest that loss of complexity is not an obligatory consequence of aging, but instead, can be optimized with appropriate intervention^37^. These interventions have been aimed at 1) augmenting the quantity and/or quality of a specific input to the postural control system, or 2) simultaneously enhancing multiple functional components of the system^37^. For example, Zhou *et al*.^38^ recently demonstrated that a shoe insole system, delivering sub-sensory vibrations to the foot soles, and thereby increasing the “input” to the postural control system, increased postural sway complexity by an average of 11% in a small cohort of healthy older adults. Moreover, this increase in postural sway complexity significantly correlated with improvement in mobility as measured by Timed-up-and-go test^38^. On the other hand, Tai Chi training is a multifaceted intervention that enhances multiple elements of the postural control system (e.g., lower extremities strength^39^, cardiorespiratory fitness^40^, and cognitive function^41^). Moreover, it has also been shown to significantly augment the complexity of standing postural sway in both healthy older adults^42^ and those suffering from peripheral neuropathy^43^, and reduce the risk of falling^44^.

In this study, falls were defined as any unintentional event in which the body came to rest on the ground or other lower level. Future work is therefore needed to determine if MSE-derived postural sway complexity can predict more specific types of falls, such as those that occur indoors or result in injury or hospitalization^45^. As the complexity in the current study is quantified using MSE only in AP postural sway due to the lack of enough signal to noise ratio in the time-series of ML direction, future studies are also needed to examine the functional importance of postural sway complexity within the ML direction. It is also of note that our current results may seem inconsistent with previous studies reporting that biological aging or disease is linked to relatively greater physiologic “complexity”^46, 47^. Khandoker *et al*.^47^, for example, reported that the complexity of minimum foot clearance time series during walking, as quantified using approximate entropy^48^ *on a single time scale*, was higher in fallers as compared to non-fallers. We know that physiological systems, including the postural control system, are regulated by numerous functional components that act across multiple tempo-spatial scales. As such, for a given physiologic time-series, estimates of “complexity” derived from single scales may be disparate from those derived and averaged across multiple scales. Costa *et al*.^49^ reported that the single-scale entropy (Scale 1) of heart beat time-series was significantly lower in healthy individuals as compared to those with atrial fibrillation (AF). On the other hand, when entropy was averaged across multiple scales, it was much lower in those with AF as compared to their healthy counterparts. This suggests that the dynamics of physiologic systems are dependent upon the measured temporal or spatial scale. Future research should therefore apply and compare these and other analytical techniques (e.g., detrended fluctuation analysis^50^) to more fully characterized postural sway dynamics. Nevertheless, the present observations indicate that multiscale complexity of standing postural sway, particularly under DT conditions, may aid in the clinical prediction of future falls in older adults. Moreover, fall-prevention strategies specifically designed to restore and enhance physiological complexity may be particularly beneficial within this population.

## Methods

### Participants

This secondary analysis was completed using data from the population-based MOBILIZE Boston Study (MBS), which aims to investigate and identify novel risk factors for falls in older adults. A complete description of the MBS study has been reported previously^17, 51^. Briefly, community-dwelling older adults aged 70 years and older who were able to walk 20 feet without personal assistance (walking aids permitted) were included. Those with terminal disease, severe vision or hearing deficits, or diminished cognitive function (i.e., Mini Mental State Examination score ≤18) were excluded. All the experimental methods and protocols were approved by the Hebrew SeniorLife Institutional Review Board (HSL IRB) and carried out in accordance with relevant guidelines. All participants provided written informed consent as approved by the HSL IRB.

At baseline, 765 eligible participants completed a home interview and were assessed for demographic, clinical and functional characteristics, including standing postural control and the Short Physical Performance Battery test (SPPB). Historical falls rate, that is, the self-reported number of falls suffered within the year prior to the baseline assessment, was also recorded.

Falls were then tracked for 48 months using monthly falls calendars and follow-up interviews. Those with incomplete falls tracking data for the entire 48 months (n = 27) were excluded from analyses.

### Assessment of standing postural control

Standing postural control was assessed by measuring postural sway (i.e., center-of-pressure, COP) fluctuations at 240 Hz with a force plate (type 9286AA, Kistler, Amherst, NY). Participants stood barefoot with feet shoulder-width apart on the force plate, which was placed with its mediolateral axis parallel to the laboratory wall. Tissue paper was placed on the force plate and chalk outlines of each foot were recorded prior to the first trial. The outline of each participant was then used throughout his/her assessment to ensure consistent foot placement across trials.

Each participant completed five, 30-second trials under two conditions: quiet standing with eyes open (i.e., single task standing, ST) and standing while performing an additional cognitive task (i.e., dual task standing, DT). Trial order was randomized and one minute of rest was given between each trial. In dual task standing trials, participants performed verbalized serial subtractions of three beginning at the number 500. If participants made five or more errors in a single trial, the test was switched to counting backwards by five from 500. In each subsequent trial, participants were asked to continue subtracting from the final number reached in the previous trial.

As previously reported, the signal-to-noise ratio (SNR) of the force plate in the laboratory was examined by comparing the COP signals recorded from a static 50 lb (22.7 kg) weight and a healthy participant^26^. We found that the SNR of the COP fluctuations in the anterioposterior (AP) direction was larger than 10 while in mediolateral (ML) direction it was smaller than 1. Therefore, we focused the current analysis of postural sway complexity on the COP time series in AP direction only (see Fig. 1).

The complexity metric was computed using MSE. Prior to calculation of MSE, empirical mode decomposition (EMD) was used to remove low-frequency trends and high-frequency noise in the raw time series, which was well-established previously^19^. Specifically, fluctuations at frequencies over 20 Hz were removed, as they are unlikely to reflect physiologically meaningful control processes. Fluctuations at frequencies less than 0.2 Hz were also removed, so as to ensure that a sufficient number of dynamic patterns occurred within the length of COP time series^19^.

EMD-filtered time series were “coarse-grained” on different scales of time to capture system dynamics. This procedure divided the time-series into non-overlapping windows of length equaling a scale factor, τ, ranging from 1 to 40 data points in this study. Thus, the coarse-grained series at the largest scale had 180 data points (i.e., 7200 points/40), which meets standard practice for obtaining reliable estimates of sample entropy^18^. Sample entropy is defined by the negative natural logarithm of the conditional probability that a time-series, having repeated itself within a tolerance *r* for *m* points (pattern length), will also repeat itself for *m* + *1* points without self-matches. The sample entropy of each coarse-grained time-series in this study was computed by choosing *m* = 2 and *r* = 15%^19^. Figure 1C showed the MSE curves generated by plotting sample entropy as a function of time scale from the two COP time-series presented in Fig. 1A and B. Finally, the postural sway complexity metric was identified as the area under the MSE curve (See Fig. 1C, “shaded region”). A larger area reflects higher sample entropy values over multiple time scales and thus, greater complexity.

In addition to postural sway complexity, several traditional measures of postural sway were computed to enable the comparison of their relationship to future falls. These measures included average sway speed (i.e., COP distance traveled in one trial divided by duration of the trial), sway area (i.e., the area of a confidence ellipse enclosing 95% of the COP fluctuation) and AP path length (total COP distance traveled in the AP direction). Since only AP MSE was used, we included AP path length to provide an additional comparison of sway in this specific direction.

### Assessment of falls

A fall was defined as unintentionally coming to rest on the ground or other lower level, not as a result of an overwhelming external hazard or a major intrinsic event. Any fall episodes not classified using this definition were reviewed by an adjudication panel^52^. Falls were tracked for 48 months using monthly post-card calendars. Participants were instructed to record the actual date when they experienced a fall on the calendar and to mail their calendars to the study center at the end of each month. Those who failed to return the calendars were contacted and interviewed by telephone to collect information on their falls. If a fall was reported on the calendar, a structured telephone interview was conducted to clarify the details of the reported falls, including the circumstances and location of the fall, whether the fall caused injury, etc.

### Statistical analysis

Analyses were performed with SAS 9.4 and JMP Pro 12 software (SAS Institute, Cary NC). Means, standard deviations (S.D.) and percentages of selected descriptive characteristics were calculated for the study sample. The Chi-Square test was used to identify the difference in dichotomous baseline variables (e.g., sex) and ANOVAs were used to determine the difference in continuous baseline characteristics between fallers and non-fallers.

Negative binomial regression was used to model the association between the baseline postural sway metrics (i.e., complexity, sway speed, sway area and AP path length) during ST or DT conditions, SPPB scores and future fall rates tracked over 48 months. Covariates for all negative binomial models included age, sex, body mass index (BMI) and historical falls rate.

Additionally, we created quintiles of the continuous postural sway complexity metric separately for ST and DT. ANOVAs and Student’s t tests were first performed to determine if selected health characteristics differed between these quintiles (Chi-Square test was used to identify the difference in sex). Negative binomial regression was then used to compare the rate of future falls between different quintiles of postural sway complexity. The quintile of highest postural sway complexity was set as the reference group. Covariates for these models similarly included age, sex, BMI and historical falls rate.

The incident rate ratio (IRR) and 95% confidence intervals (95% CI) were obtained from all the negative binomial models and the significance level for all analyses was set to p < 0.05.
